# Applying AI in the Context of the Association Between Device-Based Assessment of Physical Activity and Mental Health: Systematic Review

**DOI:** 10.2196/59660

**Published:** 2025-03-06

**Authors:** Simon Woll, Dennis Birkenmaier, Gergely Biri, Rebecca Nissen, Luisa Lutz, Marc Schroth, Ulrich W Ebner-Priemer, Marco Giurgiu

**Affiliations:** 1 Mental mHealth Lab Institute of Sports and Sports Science Karlsruhe Institute of Technology Karlsruhe Germany; 2 Department of Embedded Systems and Sensors Engineering Research Center for Information Technology Karlsruhe Germany; 3 Department of Psychiatry and Psychotherapy Central Institute of Mental Health Medical Faculty Mannheim, Heidelberg University Mannheim Germany; 4 German Center for Mental Health Mannheim Germany

**Keywords:** machine learning, mental health, wearables, physical behavior, artificial intelligence, mobile phone, smartphone

## Abstract

**Background:**

Wearable technology is used by consumers worldwide for continuous activity monitoring in daily life but more recently also for classifying or predicting mental health parameters like stress or depression levels. Previous studies identified, based on traditional approaches, that physical activity is a relevant factor in the prevention or management of mental health. However, upcoming artificial intelligence methods have not yet been fully established in the research field of physical activity and mental health.

**Objective:**

This systematic review aims to provide a comprehensive overview of studies that integrated passive monitoring of physical activity data measured via wearable technology in machine learning algorithms for the detection, prediction, or classification of mental health states and traits.

**Methods:**

We conducted a review of studies processing wearable data to gain insights into mental health parameters. Eligibility criteria were (1) the study uses wearables or smartphones to acquire physical behavior and optionally other sensor measurement data, (2) the study must use machine learning to process the acquired data, and (3) the study had to be published in a peer-reviewed English language journal. Studies were identified via a systematic search in 5 electronic databases.

**Results:**

Of 11,057 unique search results, 49 published papers between 2016 and 2023 were included. Most studies examined the connection between wearable sensor data and stress (n=15, 31%) or depression (n=14, 29%). In total, 71% (n=35) of the studies had less than 100 participants, and 47% (n=23) had less than 14 days of data recording. More than half of the studies (n=27, 55%) used step count as movement measurement, and 44% (n=21) used raw accelerometer values. The quality of the studies was assessed, scoring between 0 and 18 points in 9 categories (maximum 2 points per category). On average, studies were rated 6.47 (SD 3.1) points.

**Conclusions:**

The use of wearable technology for the detection, prediction, or classification of mental health states and traits is promising and offers a variety of applications across different settings and target groups. However, based on the current state of literature, the application of artificial intelligence cannot realize its full potential mostly due to a lack of methodological shortcomings and data availability. Future research endeavors may focus on the following suggestions to improve the quality of new applications in this context: first, by using raw data instead of already preprocessed data. Second, by using only relevant data based on empirical evidence. In particular, crafting optimal feature sets rather than using many individual detached features and consultation with in-field professionals. Third, by validating and replicating the existing approaches (ie, applying the model to unseen data). Fourth, depending on the research aim (ie, generalization vs personalization) maximizing the sample size or the duration over which data are collected.

## Introduction

### Background

Mental disorders such as depression, anxiety, or bipolar disorder remain among the top 10 leading causes of burden worldwide [1], with an estimated number of 970 million people in the world living with a mental disorder and a steady prevalence rate of around 13% [2]. The economic burden is estimated at US $5 trillion [3]. Moreover, global changes such as the COVID-19 pandemic may further boost the prevalence rate of mental health disorders [4]. Thus, the prevention and management of mental health are urgent topics. Researchers identified that social determinants of health represent the most modifiable set of targets for nonpharmacological intervention to prevent the onset of mental health disorders. One of these modifiable lifestyle behaviors that can play an evident role in the prevention and management of mental health is being physically active and performing regular exercise in daily life [5]. Earlier meta-analysis indicated that regular aerobic exercise results in moderate increases in self-reported affect [5,6]. A further review showed that the association between physical activity and mental health in young people is evident with small to moderate effects [7].

Previous studies applied traditional assessment methods (eg, self-reports and questionnaires) and analytical approaches (eg, regression analyses and 1-tailed *t* tests) and were able to unveil associations between physical activity and mental health outcomes retrospectively. A methodological aspect of earlier studies that display weaker research designs is the application of self-reports for the assessment of physical activity. Prince et al [8] compared self-reports and direct measures of physical activity across 148 studies and concluded that the measurement method may have a significant impact on the observed levels of physical activity. In particular, for example, due to retrospective biases, self-report measures of physical activity were both higher and lower than directly measured levels of physical activity. In line with technological developments, previous studies intensively used accelerometers as a passive monitoring device for capturing physical activity. The purpose of their application is variable, such as an observational tool in surveillance studies [9]; a motivational tool in interventions [10]; a way to better understand the underlying mechanisms of health, treatment, and recovery (ie, understanding the role of physical behavior in it); or as a diagnostic tool in clinical settings [11]. Using a device-based assessment of physical activity, researchers replicated the positive impact of physical activity on various mental health outcomes. For example, a meta-analysis found that higher levels of physical activity may offer prevention against the onset of depression [12], stress-related disorders [13], and psychotic disorders [14]. Furthermore, studies also indicated preliminary results that being physically inactive in terms of spending higher time in sedentary behavior is associated with an increased risk of various mental disorders [15-17]. In addition, not only observational studies but also results of randomized controlled trials have shown that physical activity interventions may reduce mental health symptoms among individuals affected by depression, stress-related disorders, and schizophrenia [18]. Most of those studies applied traditional statistical approaches, for example, analyzing the association between sedentary behavior and mood dimensions via multilevel modeling [19]. However, those approaches are limited in predicting, classifying, or detecting future conditions.

Nowadays, the ability to collect raw accelerometer data without great effort (eg, via passive continuous longitudinal measurements from wearables) and in large quantities allows the application of sophisticated artificial intelligence algorithms for the detection, classification, or prediction of mental health states and traits. In this context, the widely used term “machine learning” refers to a range of mathematical techniques that leverage the computational power to identify meaningful patterns within a large dataset [20]. The application of machine learning algorithms comprises a series of iterative processes and phases from learning and training within a single original dataset over testing in a new dataset. The use of artificial intelligence can be seen as a new methodological area with increasing awareness in health science but not yet established in clinical research and practice. Contrary to traditional approaches, which account for patterns in an original dataset, machine learning algorithms evaluate how accurately they can predict patterns and relationships in new datasets [20]. Furthermore, machine learning algorithms emphasize multidimensionality by capturing the complex interactions between multiple potential predictors and the outcome of interest. The application of machine learning can serve different purposes—some models are trained on groups to provide general predictions, classifications, or detections applicable to all individuals (ie, generalizability), while others are personalized, trained on an individual’s data to generate tailored predictions specific to that person [21].

Comparing traditional retrospective and modern analytical methods (eg, machine learning), we see a gap in both the possibilities and the performance. Passive measurement methods in the use of wearables have become established in research studies and make it possible to record enormous high-resolution datasets in everyday life. However, it is still unclear how the potential can be optimally used on the basis of new evaluation approaches. Machine learning enables new strategies to analyze huge amounts of data compared to traditional methods (eg, retrospective vs real-time analysis of data, analysis of new datasets with already existing models without new training in less time, and possible forecasting of future trends and events). To get an overview of the status quo, we summarized studies that integrated passive monitoring of physical activity data measured via wearable technology in machine learning algorithms for the detection, prediction, or classification of mental health states and traits.

### Objective

Although the application of machine learning approaches in health science increased over the last years [22], we are not aware of a previous work that integrated passive monitoring of physical activity data measured via wearable technology in machine learning algorithms for the detection, prediction, or classification of mental health states and traits. Thus, the aim of the review is (1) to provide a comprehensive overview of studies that combined machine learning approaches based on accelerometer measures with mental health outcomes; (2) to summarize the results of the selected studies, the applied machine learning methods, and the used wearable technologies; and (3) to rank and discuss quality aspects of the studies.

## Methods

### Overview

This study followed the PRISMA (Preferred Reporting Items for Systematic Reviews and Meta-Analyses) reporting guidelines [23] and was registered in the PROSPERO international prospective register of systematic reviews (CRD42023436926). The PRISMA checklist can be found in Multimedia Appendix 1.

### Search Strategy and Study Selection

To identify relevant publications, we used a search string that included terms for (1) machine learning, (2) physical activity, and (3) wearables (Multimedia Appendix 2). Publications were searched from 1970 to March 2023 using the following databases: EBSCOhost, IEEE Xplore, PubMed, Scopus, and Web of Science.

All papers were imported to a reference manager, Citavi library (Citavi, version 6.14; Swiss Academic Software GmbH). After merging all duplicates first electronically and afterward manually, the study selection process included 3 screening phases for eligibility. In the first phase, a minimum of 2 reviewers (SW, MG, and RN) independently screened the titles of the publications. Papers were only excluded if all reviewers categorized a paper as not eligible for review purposes. In the second phase, a minimum of 2 reviewers independently screened the publications’ abstracts (SW, MG, and RN) to determine whether a full-text review was warranted. Only those papers moved to the next phase if a minimum of 1 reviewer (SW, MG, or RN) categorized it as eligible. In the third phase, the full texts of the remaining papers were assessed for eligibility by 4 reviewers (SW, MG, RN, and LL). Each paper was screened independently by at least 2 reviewers (SW, MG, RN, or LL). Discrepancies in screening were resolved by a third review and if unanimously a fourth review. If there was still no agreement, the papers were discussed until a consensus was reached. There was direct agreement (after 2 ratings) in 52% (73/140) of the studies, a third rating was necessary in 29% (41/140) of the studies, and a fourth rating was needed in 19% (26/140) of the studies. After the fourth rating, 3 studies needed further discussion to decide whether to consider the papers in the review. Reviewers were not blinded to author or journal information.

### Inclusion and Exclusion Criteria

We included peer-reviewed, English-language publications that met the following criteria: first, studies were conducted under real-life scenarios. Second, raw accelerometer measures were collected with wearables or smartphones. Third, machine learning methods were used to process the data. Fourth, input for the machine learning algorithm must include physical activity measurements. Fifth, the outcome must be mental health–related. All other papers were excluded, for example, technical descriptions and protocols, studies with synthetically generated data, studies with animals, or not mental health–related such as pure human activity recognition.

### Data Extraction

In total, 3 authors (SW, RN, and LL) independently extracted data. For every study, one author (SW, RN, or LL) extracted data from the paper, and another author (SW, RN, or LL) complemented the extracted data with additional information. Occurring discrepancies were discussed until a consensus was reached. The following study details were extracted: author, year, location, population information (sample size, mean age of participants, percentage of male population, ethnicity, and special population group), study protocol (measurement period, type, and environment), study purpose (mental health outcome), used data (wearable, wearing position, physical activity recording, and additional sensors used), machine learning (algorithms, software, feature description, and classifier performance), study conclusion, funding, and conflict of interest information.

### Quality Assessment and Feasibility

Since no quality assessment tool was found that covered all quality aspects of interest, we modified a previously used version [24] based on a combination of the appraisal tool for cross-sectional studies and the Newcastle-Ottawa Scale for longitudinal studies. We adopted the mentioned quality assessment tools and modified them to fit our specific purposes by complementing them with our own categories and items. A total of 9 categories were selected, and each category scored between 0 and 2 points, resulting in a maximum achievable score of 18 points. In particular, we defined scoring rules to get points (Multimedia Appendix 3). For example, in the category “missing data management and dataset balancing” studies received 0 points if no information was provided, 1 point if the handling of missing data is described, and a further point if an imbalanced dataset problem is addressed. Finally, we split the points into 3 classes: low (0-6 points), moderate (7-12 points), and high (13-18 points).

## Results

### Overview

**Figure 1:**
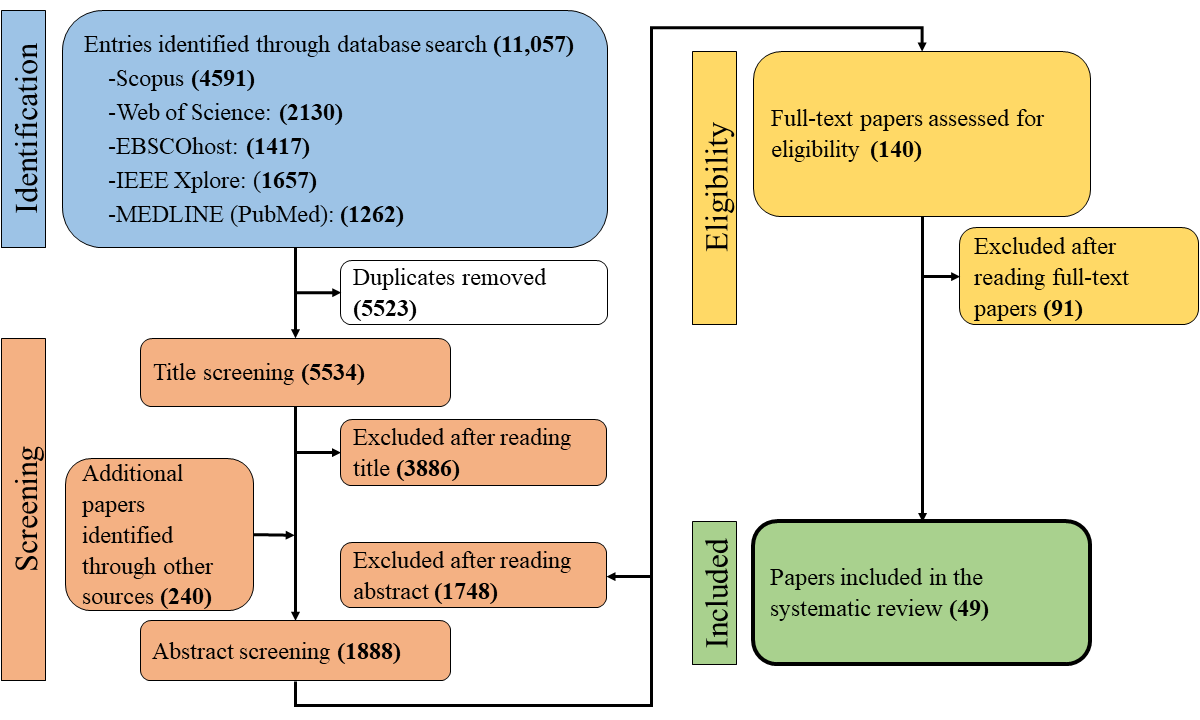
PRISMA (Preferred Reporting Items for Systematic Reviews and Meta-Analyses) flowchart.

Out of 11,057 records, we removed 5523 duplicates. The remaining 5534 papers were screened by title, and a further 3886 papers were excluded. We included another 240 papers from different sources (eg, citation screening). After screening all abstracts, 140 papers remained. After reading all full texts, 49 publications were eligible for the current systematic review. The process can be seen in Figure 1.

All 49 selected studies with corresponding extracted data points are listed in Multimedia Appendix 4 [25-73]. A brief overview of the studies can be found in Table 1. Numbers reported must not always add up to 49, as some studies belong to more than 1 subcategory.

**Table 1:** Overview over all included studies and extracted data.

Category and subcategory		Values (N=49), n (%)
**Publication year**		
	2016-2019	16 (33)
	2020-2023	33 (66)
**Study location**		
	Africa	0 (0)
	Asia	20 (41)
	Europe	12 (24)
	North America	16 (33)
	Australia or Oceania	0 (0)
	South America	1 (0)
**Sample size**		
	≤20	12 (24)
	21-50	11 (22)
	51-100	12 (24)
	101-1000	12 (24)
	>1000	2 (0)
**Study duration (days)**		
	≤1	8 (16)
	2-7	10 (20)
	8-14	9 (18)
	15-31	6 (12)
	32-90	8 (16)
	>90	5 (10)
	NR^a^	3 (1)
**Study setting**		
	Free living	34 (69)
	Laboratory	7 (14)
	Clinical setting	5 (10)
**Mental health outcome**		
	Depression	14 (29)
	Emotions	5 (10)
	Fatigue	2 (0)
	Mood	5 (10)
	State of mind	2 (0)
	Stress	15 (31)
	Other	9 (18)
**Sensor class**		
	Commercial wearable	22 (45)
	Scientific sensor carrier	18 (37)
	Smartphone	13 (27)
**Sensor position**		
	Wrist	33 (67)
	Chest	3 (1)
	Other	5 (10)
	NR	10 (20)
**Machine learning used**		
	Decision tree	8 (16)
	K-nearest neighbor	8 (16)
	Linear regression	15 (31)
	Long short-term memory	8 (16)
	Multilayer perceptron	6 (12)
	Neural network	17 (35)
	Random forest	28 (57)
	Support vector machine	18 (37)
	Extreme gradient boosting	18 (37)
	Other algorithms	15 (31)
	NR	1 (0)

^a^NR: not reported.

### Participant and Study Characteristics

The paper’s publication dates ranged from 2016 to 2023, with nearly half of them being published after 2020 (n=22, 45%). The studies are from a total of 21 different countries, leading in the list is the United States with a total of 15 studies followed by the Republic of Korea with 5 studies. The number of participants ranged from 2 to 4612, with a mean number of 232.68 (SD 310.86). Almost half of the studies (n=23, 47%) recruited less or equal to 50 participants. Only 14 studies had more than 100 participants. Only 29 studies reported information about the age and sex of the study participants. Almost all of these studies (n=26) focused on adults aged between 20 and 50 years, whereas 2 studies examined older people with a mean age of 70 (SD 10) years, and 1 study examined teenagers between the age of 12 and 20 years. In 21% (n=6) of these studies, less than one-third were male participants. In total, 17 studies had a sex distribution between one-third and two-thirds of male participants. In 6 studies, more than two-thirds of the participants were male. Convenient samples such as college or university students were recruited in 13 studies, and 8 studies recruited patients with a specific illness or condition and a distinct control group. Almost all studies were observational studies (n=45, 92%), and the remaining studies were experiments. The duration of the studies varied from less than 1 day to 365 days, with a mean of 48.04 (SD 54.10) days. A total of 27 studies had a duration of 14 or fewer days, with 6 studies having less than 1 day. Only 6 studies had 3 months or more of data recording. Most studies (n=34, 69%) were carried out in free-living conditions, while 11 reported a clinical or laboratory environment.

### Mental Health

The focus of the studies had to be connected to a mental health state or trait. In total, we identified 14 different outcomes. A total of 31% (n=15) of the studies had stress as their main focus, while 29% (n=14) examined depression. Further, we identified 5 studies each on mood and emotion outcomes. The other studies investigated topics like the state of mind, posttraumatic stress disorder, fatigue, or different anxiety disorders. Almost all studies used an initial onboarding phase with different questionnaires or ecological momentary assessment procedures. Across all studies, 14 studies were concerned with classification into different groups (eg, classifying a serious mental illness participant group from a control group or classifying participants into different severe depression levels), while 16 studies focused on detecting individual characteristics (eg, different emotions). The remaining 19 studies focused on predicting future conditions (eg, predicting the presence and severity of a depressive state or predicting a future stress rating).

### Physical Activity Assessment Via Wearables

All of the included studies collected sensor information from either a wearable or a smartphone or in 4 cases both. Of all studies, 22 used commercial-grade wearables (eg, Fitbit fitness tracker), 18 studies used scientific sensors (eg, ActiGraph) to record sensor measurements, and 13 studies used smartphones. The most used wearing position was the wrist (n=33, 67%), followed by 8 studies that used other wearing positions such as chest, hip, thigh, or finger-worn sensors. In total, 10 studies had no specific wearing position because they used smartphone recordings as used in everyday life. Physical activity was always measured with triaxial accelerometer sensor measurements. Only 5 studies used exclusively accelerometer sensor values as input, whereas all other studies used a minimum of 1 additional sensor. In particular, 17 studies used heart rate recordings, 4 studies used electrocardiography and 2 studies used heart rate variability recordings. Furthermore, 12 studies used location recordings, primarily acquired via GPS, and 10 studies used electrodermal activity, also mentioned as galvanic skin response or electrodermal response measurement recordings. Gyroscope as an additional body movement sensor was included in 9 studies.

According to the selection of raw measurements or preprocessed features, 27 of the studies used steps as physical activity features, 21 used raw accelerometer values, and 13 studies did not specify how they measured activity or used activity classification. Further, 7 studies used energy expenditure (eg, metabolic equivalent of task or calories) as a measure of physical activity.

### Machine Learning

The features extracted from the recorded sensor measurements and used by the evaluated machine learning algorithms were reported by 63% (n=31) of all studies. Only 27 studies reported a feature evaluation. The top 10 features were widely spread, and the consensus regarding what are the best 10 features for a specific use case was very low. Most studies explored more than 1 machine learning algorithm and compared the monitored performance. More than half of all studies (n=28, 57%) analyzed the use of random forest, followed by support vector machines (n=18, 37%), different neural networks (eg, deep neural network, convolutional neural network, and recurrent neural network; n=17, 35%), linear regression (n=15, 31%), and extreme gradient boosting (XGB; n=13, 27%). Overall, 39 studies reported their best-performing classifier led by XGB (n=10, 26%), followed by random forest (n=9, 23%) and support vector machine (n=5, 13%). When it comes to performance values, 39 studies reported the performance achieved by their used machine learning algorithm. The top reported performance value was accuracy (n=35, 90%), followed by sensitivity (n=25, 64%), precision (n=20, 51%), and *F* value (n=18, 46%).

In total, 22 studies reported the used programming language or used packages. In particular, 12 studies used Python (Python Software Foundation) as a programming language, followed by 7 studies that used R (R Foundation for Statistical Computing). Only 39 studies reported some performance values, and 15 of them reported only 1 or 2 performance values (mostly only accuracy or area under the receiver operating characteristic curve values). The most mentioned limitations were a small sample size (n=20, 41%), lack of diversity in the sample (n=14, 29%), or a low number of data points (n=10, 20%).

**Figure 2:**
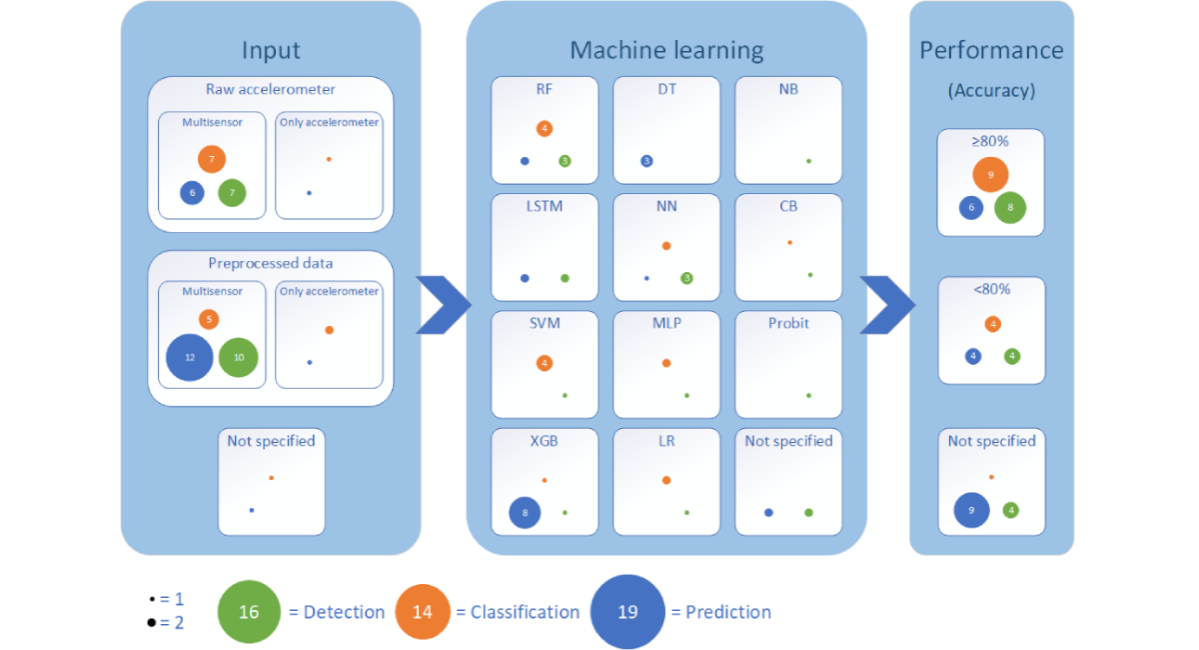
Overview of the input, machine learning approaches, and performance differentiated by the study aims. Notably, some studies appear multiple times due to different input configuration. CB: cat boost; DT: decision tree; LR: linear regression; LSTM: long short-term memory; MLP: multilayer perceptron; NB: naïve Bayes; NN: neural network; RF: random forest; SVM: support vector machine; XGB: extreme gradient boosting.

Figure 2 presents a summary of the input, machine learning approaches, and performance differentiated by the study aims, namely, detection, prediction, or classification of mental health states or traits. The most significant visual aspects are described in the following. Obviously, only 5 studies used accelerometer measures as input signals, whereas most studies combined accelerometer measures with additional sensor signals such as gyroscope or heart rate. Nearly half of the studies that focused on the prediction of mental health outcomes applied XGB as a machine learning algorithm. In contrast, studies that focused on detection reported a variety of 11 different approaches. Nearly half of the studies that focused on the prediction of mental health outcomes and used XGB as a machine learning model did not report the accuracy. Nearly two-thirds of all studies that focused on the classification reported overall high accuracy (≥80%). A more specific view on the different mental health outcomes revealed, for example, that raw data were more frequently used for the classification of depression, and the classification accuracy is quite promising. In comparison, when it comes to stress, the distribution of input data, machine learning, and accuracy performance is more balanced. For more details on the distribution of outcomes and specific details about the 2 most studied mental health outcomes, depression and stress, see Multimedia Appendix 5.

### Study Quality

**Figure 3:**
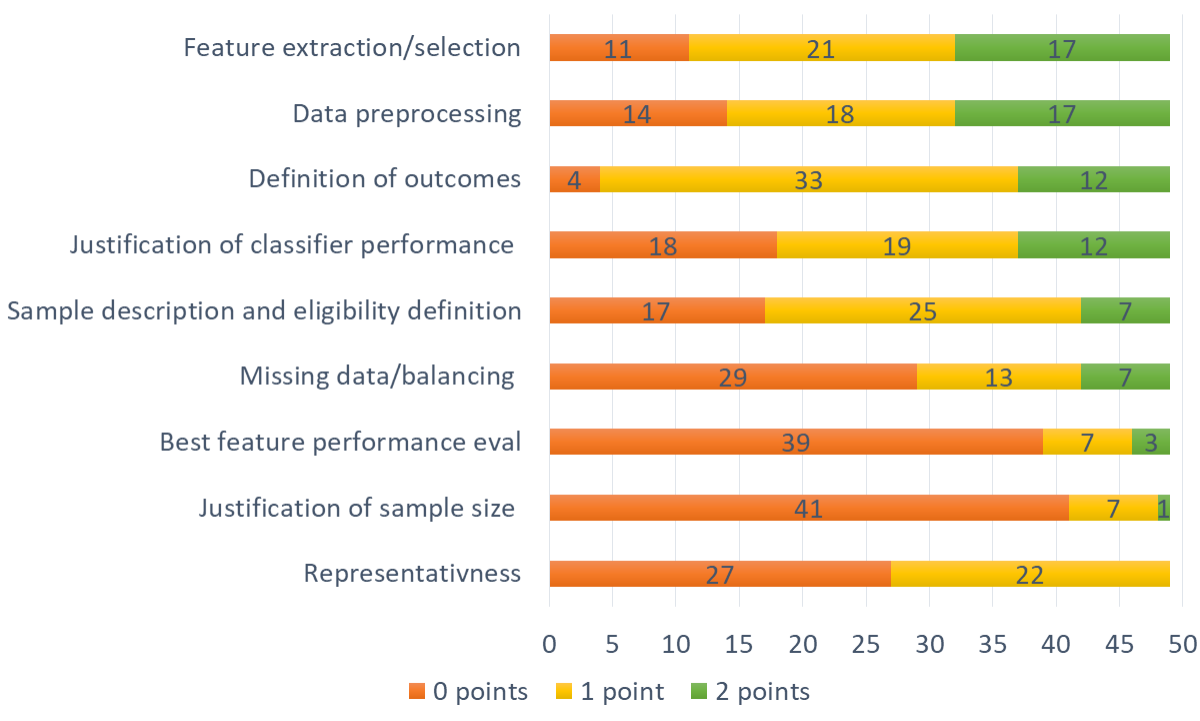
Quality of the papers sorted by categories.

We rated all 49 studies in the 9 categories and classified them into the specified quality class. We classified 21 studies as low quality, 27 studies with moderate quality, and only 1 study with high quality. From another perspective, only 10 studies reached half of the points or more. On the positive side, almost all studies defined the outcome to achieve, but 4 studies did lack even a description of what they wanted to achieve. In the “representativeness” category, no study got 2 points mostly because they did not provide their implementation or data or did not have an independent validation (eg, testing the implemented model with another independent dataset). In the category “justification of sample size,” only a total of 9 points (of possible 98 points) was scored. Reporting the sample size is important, as small sample sizes could lead to inaccurate results and therefore to drawing false conclusions [74]. On average, the papers were rated with 6.47 (SD 3.1) points; therefore, the overall quality of the reviewed studies can be rated moderate to low. Figure 3 shows the distribution of points in the individual categories.

## Discussion

### Principal Findings

In our review, we included a total of 49 studies that applied machine learning methods based on accelerometer measures via wearable technology to predict, detect, or classify mental health states or traits. According to the aims of our review, we can summarize the following main results: first, studies focused on different mental health objectives (prediction, classification, or detection of affect or mood, anxiety, depression, anorexia nervosa, emotions, fatigue, posttraumatic stress disorder, state of mind, and stress). The 2 most frequently studied outcomes were stress with 15 studies and depression with 14 studies. Second, we identified heterogeneity in the used machine learning methods. In particular, across all studies, 30 different machine learning algorithms were used and integrated human acceleration data either from smartphones or wearables. Third, the top 10 studies with the highest accuracy and 8 of the top 10 studies with the highest precision used raw accelerometer measurements instead of already preprocessed data. Fourth, the quality of the studies can be judged as low to moderate. Although the studies pursued different purposes and followed different methodological approaches, obvious limitations become apparent that merit further consideration.

### Raw Measurements and Features

As an inclusion criterion for the selection of studies, we set the requirement that either raw accelerometer measurements or at least preprocessed parameters from raw accelerometer measurements were included as an input signal to the machine learning algorithm. In total, 22 studies used raw accelerometer measurements, whereas 27 studies used preprocessed data such as steps or dimensions of energy consumption. According to Fedor et al [22], raw measurement describes the data recorded by wearables or smartphones (eg, accelerometer, gyroscope, location, and heart rate) and can provide direct insights about general mental well-being. For example, an approximation of stress detection can be associated with heart rate or heart rate variability [75].

When focusing on those studies that reported machine learning performance values, the top 10 studies that reported high accuracy used raw accelerometer measurements as input. Moreover, focusing on the performance classifier precision, 8 of the top 10 studies also used raw accelerometer measurements. When looking at sensitivity and *F* value, 6 of the top 10 studies used again raw accelerometer measurements. This suggests the assumption that using raw accelerometer measurements instead of preprocessed data provides better overall performance. Thus, whenever possible, to ensure the objectivity of the input data, we encourage future research efforts to develop machine learning algorithms based on raw accelerometer measurements rather than preprocessed data. In other words, if algorithms are based on preprocessed data, decisions have already been made that may influence the results [76]. There is no consensus about how the raw data should be processed optimally and harmoniously. In particular, different possibilities for processing raw accelerometer data into metrics or parameters are presented in the literature, for example, counts [77], movement acceleration intensity [78], Euclidian norm minus one [79], or mean amplitude deviation [80]. Furthermore, 20 of all included studies integrated data from commercial-grade wearables such as Fitbit or Garmin. This brings up further challenges, as researchers often do not have access to raw accelerometer measurements of consumer wearables, and they do not have access to the “black-boxed” algorithms either [76]. Optimally, researchers should have access to raw accelerometer measurements or high-resolution feature data that are derived from the raw data. Furthermore, at the very least, comprehensive details of algorithms as well as the version of the device and software should be made transparent. In this context, transparency is a requirement for reproducibility, comparability, stacking of datasets, or creating norms. In line with the mentioned challenges, a significant number of wearables are not validated through high-quality protocols [81,82].

Nearly all studies (n=39) integrated additional measurements next to accelerometer measurements such as heart rate, location, electrodermal activity, or ambient light for the application of the machine learning algorithm. The idea behind this approach might be to add supplementary information to increase the chance to identify patterns. However, we suggest adding only meaningful data to the algorithm based on empirical evidence [22]. Besides the aim to maximize the most accurate prediction, detection, or classification of mental health states or traits based on several raw measurements, it might also be interesting to identify the isolated impact of, for example, raw accelerometer or heart rate measurements on mental health outcomes. We expect an increase in studies applying a machine learning approach in the future and thus the opportunity to tackle these issues.

Although we prefer raw measurements of sensors, these are mostly not expressive enough to gain insights into specific behavior. To gain meaningful information from raw measurements, extracting the so-called low-level features is necessary. For example, wearables worn on the wrist collect linear acceleration via an accelerometer sensor. Through applying feature extraction algorithms to the raw acceleration measurements, they can be transformed into, for example, step count [83] or movement intensity [80].

In total, 18 studies did not report the used features, which hinders the possibility of replicating the application of the described machine learning model. Of the 31 studies that reported the used features, only 23 mentioned their feature selection process, which includes feature evaluation as well as describing the importance of and influence of a feature on the outcome. Feature evaluation is essential to gain insights into which features impact the outcome and what may be correlated to the studied mental health condition [84]. We strongly recommend reporting the used feature selection process and the final feature subset used in the model, especially to transparently explain how the results are achieved.

Additionally, not the most influential features should be selected but the ones highly correlated with the studied outcome. Therefore, consultation with domain experts is highly recommended to ensure that the calculated features contain the necessary information for the identification, classification, or prediction of the targeted mental health condition. Experts in the field recommend that when performing machine learning analyses, 2 assumptions are made, namely, first, the desired outputs of the data can be generated, given the input data; and second, the available data contain the necessary information to learn the desired output [22,84].

In general, the use of low-level features is essential for the application of machine learning, as it joins raw measurements into more meaningful values. A further step is the aggregation of calculated feature values into the so-called high-level features or high-level information [22]. This high-level information can combine multiple low-level features to reflect different behavioral patterns (eg, physical activity or sleep) [22,85]. We found that none of the reviewed studies integrated high-level information, which can be crucial to explain certain behaviors. For example, some studies used features like “hours of sleep” for depression detection or severity classification, but looking at only hours of sleep could not be enough. In particular, Riemann et al [86] provided an overview of the complex relationship between sleep and depression, indicating that several sleep parameters such as sleep stages or sleep efficiency are also related to depression. Therefore, combining multiple features into high-level features might be necessary to get a clearer picture. Another example is the correlation between stress and physical activity. Stress might be associated with reduced physical activity [87]. However, reduced physical activity can have various reasons besides stress (eg, illness or injury or bad weather conditions). In contrast, Smets et al [88] showed that a combination of different physiological signals (eg, electrodermal activity or pulse rate variability) and contextual information (eg, temperature and location) can also be used to predict stress. Therefore, combining both approaches might improve stress detection.

We recommend using different combinations of features depending on the targeted objective. Future studies should focus on crafting optimal feature sets for distinct detection, classification, or prediction of the various mental health states and traits rather than using many individual detached features.

### Machine Learning

While classic programming transforms input data via previously defined rules into information-enriched output data, machine learning tries to find these rules (model) that describe the correlation or connection between input data and given output [89]. The more data we have to train our rule set, the better and more precise the machine learning model can predict, classify, or detect the outcome of unknown input data. Furthermore, an additional effect of a larger dataset for training is a higher generalizability of the model. Our included studies varied widely in terms of aim and rationale. However, some methodological and machine learning–specific issues affect all studies and have an impact on the quality that merits further consideration.

First, when collecting data in studies, 2 main dimensions can increase the size of the dataset: the sample size and the duration over which data are collected. A longer duration helps collect more data points per person and increase the potential to personalize results (ie, within-subject approach), and larger sample sizes help build a more diverse dataset (ie, between-subject approach) [90]. Nevertheless, both dimensions are important to obtain meaningful output when using machine learning. Depending on the use case, however, 1 dimension might be crucial.

For detection or classification purposes, a broad and diverse sample is important for generalization [91]. However, only 2 of 13 studies addressing detection and only 4 of 14 studies focusing on classification had a sample size greater than 100. Overall, almost half of all studies (n=23, 47%) recruited less or equal to 50 participants. Only 14 studies recruited more than 100 participants. A further problem occurring in 11 of 27 studies is the acquisition of participants from a particular group of people (eg, students), which also does not play in favor of generalization.

The duration of data acquisition can play a role as well in detection or classification. Fedor et al [22] show that a recording of 16 days of physical activity and depression level might not be enough to detect an association between physical activity and depression, whereas a duration of 6 weeks may show an association. When predicting trends or forecasting events ideally, a long duration for data collection is envisaged. As seen in the aforementioned example of Fedor et al [22], trends or correlations might not be visible in short-duration datasets. In total, 9 of 19 studies examining prediction or forecasting reported a study duration of 30 days or longer, and 7 of 19 studies only reported 14 or fewer days down to even 1 day. This can mean that correlations did not lie within the analyzed period and dampened the overall performance results reported.

All included studies implemented a minimum of 1 supervised machine learning algorithm for their application. When implementing machine learning models with supervised learning, splitting your primary dataset into different partitions for training and testing is common [84]. The performance measure of the developed model is thus only valid for that particular primary dataset. For use in the clinical area, further testing is needed to ensure that the measured accuracy holds when applying the model to unseen data. Therefore, large and precisely labeled datasets from diverse populations are required [22]. None of the reviewed studies used external supplementary datasets to validate their implemented models and presented performance values. Additionally, none of the studies used different wearables—particularly studies using commercial wearables—that generate the same sensor measurements and features to validate their results. We recommend not only replicating and validating the developed models with independent datasets but also incorporating other wearables that produce the same measurements and features as the original device.

As seen in the analyzed studies, machine learning enables the detection, classification, and prediction of mental health conditions from either raw acceleration measurement or preprocessed physical activity data (eg, steps). In most studies, additional supplementary sensors are used to find possible patterns in the measured data typical for various mental health conditions. Analyzing the influence of the different sensor measurements on the outcome can help strengthen the developed models. However, only a few of the studies compared the capabilities of their machine learning models with different subsets of the recorded measurements. As a future task, not only implementing machine learning models from already collected data but also testing them in real-time and real-world scenarios optimally with live sensor measurements can help collect more insights on how to improve and reinforce the models and how to interpret the outcome [85,92].

### The Future: Clinical State

The use of machine learning is becoming increasingly popular when it comes to analyzing large datasets. Machine learning is also being used more and more frequently in sensitive areas, for example, when analyzing health-related data (eg, physical activity and sleep) [93,94]. Considerable progress has been made in some areas of application, leading to high-level information that already enables more valuable insights. Nevertheless, the advancements made are not sophisticated enough to be considered clinically relevant [22,95,96]. For validation and evaluation of developed algorithms, large datasets are needed, which are not available yet. Additionally, large studies are necessary to validate the developed applications and their reported outcomes.

Another factor influencing the outcome of the developed machine learning model is the respective context. For example, a drop-off in physical activity of a person can be a symptom of depressive disorder but can also be the result of a physical injury or bad weather conditions. Only a few studies included contextual information in the form of features in their implementation and training of their model. Here, next to passive monitoring of biosignals or geolocation, the collection of self-reported information via ecological momentary assessment is a valuable addition for deeper insights into contextual and latent factors [97,98].

For some mental health conditions, we identified only one study, such as, for posttraumatic stress disorder [25], social anxiety disorder [26], or anorexia nervosa [27]. To finally conclude, whether the mentioned conditions can be meaningfully recognized with machine learning models via physical activity and optional supplementary sensor measurements is doubtful. Therefore, consultation with in-field professionals is inevitable to assess the feasibility and to evaluate the results. Further, there are not enough studies of some of the investigated mental health conditions to be able to make a reliable statement about the significance of the results.

It should also be considered whether the mental health condition under investigation can be studied with a generalized model at all or if a personalized model needs to be developed for each individual [99]. Measuring the same value for 2 different people could mean different things. Even within 1 person, the meaning of the measurement can change over time. Therefore, huge datasets with diverse populations are necessary for development before these models are included in clinical practice.

As we get closer and closer to implementing new machine learning applications in clinical practice, studies should also focus on the cost-effectiveness of their developed solutions compared to traditional approaches. The initial costs of implementing machine learning applications are high due to the enormous amount of high-quality data needed to train and test meaningful models. After initial training, the costs decrease, as base models need less data for fine-tuning for, for example, personally tailored treatment. First studies have shown that the implementation of machine learning algorithms might be cost-effective not only when it comes to predictive analytics for early disease detection or prediction but also in the areas of remote health care and monitoring or in the management of personalized treatment (eg, maintaining medication plans) [100,101].

In conclusion, the developments in the field of mental health that use machine learning for detection, classification, or prediction are not sophisticated enough for use in the clinical area. In the near future, first applications will assist diagnosis and treatment of selected mental health conditions, but automatically estimating the full clinical state of a patient will take more time and studies [22,102]. All assessed studies explore small parts of the complex field of mental health care and can be considered the first steps toward more digital and intelligent health care systems. These studies can be seen as the first “proof-of-concept” applications in the area of digital health care applications. Despite all this, there are already initial endeavors to use machine learning applications in clinical practice. One example is analyzing electronic health records with the help of machine learning for improving disease phenotyping for differential diagnosis of major depression or predictive modeling of depression and anxiety [103-105]. In another example, machine learning is used to track the clinical state via speech analysis [106]. However, there also remain not only technical challenges but also questions about health equity, data privacy, and security and a new role of health care professionals [107].

Even though the full potential of artificial intelligence has not been reached so far, some examples demonstrate what can be achieved in the foreseeable future. Masud et al [28] developed a system to monitor daily live activities for estimating the depression score (16-item Quick Inventory of Depressive Symptomatology) as well as the depression severity level. The system has the potential to identify depression and classify the severity by assessing day-to-day activities. This enables passive monitoring and can help provide immediate health care when depression is detected. Another study by Rozet et al [29] developed models for predicting individual stress ratings. Different approaches were analyzed, more generalized nomothetic approaches and more individualized ideographic approaches. It was demonstrated that, initially, the ideographic model performed better, but with ongoing data collection, the nomothetic model surpasses it, highlighting the importance of more personalized models because of the heterogeneity of the importance of the predictors on stress. We are expecting an increase of studies that apply machine learning techniques in the area of lifestyle factors (eg, physical activity) and mental health in the future. With the increase in studies and, above all, the development of homogeneous study protocols and methods for reporting machine learning performance, meaningful meta-analyses can be conducted. Ultimately, individual participant data meta-analyses can be carried out in the future.

### Strengths and Limitations

The main strength of this study is that it focuses on several mental health conditions examined with machine learning and the possibility to detect, classify, or predict aspects of it based on wearable signals. This provides a broad overview of the possibilities of generating valuable information with the measurements of wearables in the field of mental health. Of course, there are several limitations. First, some relevant studies may have been missed because they did not appear in our search due to missing key terms in the specified search criteria. Additionally, only English-language search results were considered. Additionally, due to the fast development of machine learning applications in research, new studies might have been published after the initial search. Second, study quality was assessed using a combination of the appraisal tool for cross-sectional studies and the Newcastle-Ottawa Scale for longitudinal studies already used by De Angel et al [24] but further complemented with own categories in line with core principles, recommendations, and expert statements [24,84,108,109]. Third, due to the large heterogeneity of included studies (eg, different outcomes, different study designs, and different reported performance values), our systematic review provides no quantitative synthesis of the results in the form of a meta-analysis, as this would not allow any meaningful interpretation.

### Future Directions and Conclusions

Our review indicated that the application of machine learning in the context of the association between physical activity and mental health arose over the last couple of years, thus showing the relevance in research. The detection, classification, and prediction of mental health states and traits using machine learning based on wearable signals have shown to be a promising approach. Not only are wearables widely used and easy to operate in today’s society, but also they can provide valuable insights about nontrivial aspects like physical activity or sleep. Using machine learning models is a promising next step to gain even more high-level information. Although there are serious pitfalls, which need to be mastered before, these applications can be included in clinical practice. First, researchers should use huge amounts of diverse datasets to improve and enhance the capabilities of their machine learning models. Second, independent evaluation and validation are inevitable to ensure maximum stability and to strengthen the meaningfulness. Third, researchers should consult a domain professional for the development as well as evaluation of the application. Fourth, the research community should agree on well-formulated guidelines for reporting the analysis of the developed application to guarantee a maximum of transparency. Fifth, researchers should train the machine learning models whenever possible with data that are preprocessed as little as possible.

This review of state-of-the-art applications for assessing mental health states shows that the use of wearable data with the help of machine learning can indeed provide valuable information, but various aspects of the development and reporting process need to improve especially in such a sensitive field as mental health.

## Appendix

Multimedia Appendix 1 PRISMA (Preferred Reporting Items for Systematic Reviews and Meta-Analyses) checklist.

Multimedia Appendix 2 Search terms used in EBSCOhost, IEEE Xplore, Scopus, PubMed, and Web of Science databases.

Multimedia Appendix 3 Quality assessment categories and scoring details.

Multimedia Appendix 4 Overview of all included studies and respective extracted information.

Multimedia Appendix 5 Overview of input, machine learning, and outcome or performance.

## Abbreviations

PRISMA: Preferred Reporting Items for Systematic Reviews and Meta-Analyses
XGB: extreme gradient boosting
