# Supplementary material for: Applying AI in the Context of the Association Between Device-Based Assessment of Physical Activity and Mental Health: Systematic Review
Source: JMIR Mhealth Uhealth. 2025 Mar 6;13:e59660. doi: 10.2196/59660 (PMC11926455; doi:10.2196/59660)
Supplement: Multimedia Appendix 2 [file mhealth_v13i1e59660_app2.docx]

| **EbscoHost** | ("machine learning" OR "deep learning" OR "neural net*" OR "reinforcement learning" OR "artifical intelligence")  AND  ("physical activity" OR "physical fitness" OR walking OR "exercis*" OR "energy expenditure" OR "energetic arousal" OR "step-count" OR "locomotive activity" OR "ambulatory movement" OR "activity intensity" OR "free-living condition" OR locomotion OR "activity patterns" OR sport OR "everyday activities" OR "free-living activites" OR "sedentar*" OR sitting OR "sedentary behaviour" OR "body posture" OR "physical behav*" OR "physical inactivity")  AND  ("wearable" OR "activity monitor*" OR "activity tracker" OR "fitness tracker" OR "smartphone" OR "smartwatch" OR "smart device" OR "acceleromet*" OR "motion sens*" OR "actigraph") |
| --- | --- |
| **IEEE Explore** | ("machine learning" OR "deep learning" OR "neural net*" OR "reinforcement learning" OR "artifical intelligence")  AND  ("physical activ*" OR "physical fitness" OR walking OR "exercis*" OR "energy expenditure" OR "energetic arousal" OR "step-count" OR "locomotive activity" OR "ambulatory movement" OR "activity intensity" OR "free-living condition" OR locomotion OR "activity patterns" OR sport OR "everyday activities" OR "free-living activites" OR "sedentar*" OR sitting OR "sedentary behaviour" OR "body posture" OR "physical behav*" OR "physical inactivity")  AND  ("wearable" OR "activity monitor*" OR "activity tracker" OR "fitness tracker" OR "smartphone" OR "smartwatch" OR "smart device" OR "acceleromet*" OR "motion sens*" OR "actigraph") |
| **Scopus** | TITLE-ABS-KEY("machine learning" OR "deep learning" OR "neural net*" OR "reinforcement learning" OR "artifical intelligence")  AND  TITLE-ABS-KEY("physical activ*" OR "physical fitness" OR walking OR "exercis*" OR "energy expenditure" OR "energetic arousal" OR "step*" OR "locomotive activ*" OR "ambulatory mov*" OR "activity intens*" OR "free-living condition" OR locomotion OR "activity pattern*" OR sport* OR "everyday activ*" OR "free-living activ*" OR "sedentar*" OR sitting OR "sedentary behav*" OR "body posture" OR "physical behav*" OR "physical inactiv*")  AND  TITLE-ABS-KEY("wearable" OR "activity monitor*" OR "activity track*" OR "fitness track*" OR "smartphone*" OR "smartwatch*" OR "smart device" OR "acceleromet*" OR "motion sens*" OR "actigraph*") AND ( LIMIT-TO ( DOCTYPE,"ar" ) OR LIMIT-TO ( DOCTYPE,"cp" ) ) AND ( LIMIT-TO ( LANGUAGE,"English" ) ) |
| **PubMed** | ((("machine learning"[Title/Abstract] OR "deep learning"[Title/Abstract] OR "neural net*"[Title/Abstract] OR "reinforcement learning"[Title/Abstract] OR "artifical intelligence"[Title/Abstract]))  AND  (("physical activ*"[Title/Abstract] OR "physical fitness"[Title/Abstract] OR walking[Title/Abstract] OR "exercis*"[Title/Abstract] OR "energy expenditure"[Title/Abstract] OR "energetic arousal"[Title/Abstract] OR "step*"[Title/Abstract] OR "locomotive activ*"[Title/Abstract] OR "ambulatory mov*"[Title/Abstract] OR "activity intens*"[Title/Abstract] OR "free-living condition"[Title/Abstract] OR locomotion[Title/Abstract] OR "activity pattern*"[Title/Abstract] OR sport*[Title/Abstract] OR "everyday activ*"[Title/Abstract] OR "free-living activ*"[Title/Abstract] OR "sedentar*"[Title/Abstract] OR sitting[Title/Abstract] OR "sedentary behav*"[Title/Abstract] OR "body posture"[Title/Abstract] OR "physical behav*"[Title/Abstract] OR "physical inactiv*"[Title/Abstract])))  AND  (("wearable"[Title/Abstract] OR "activity monitor*"[Title/Abstract] OR "activity track*"[Title/Abstract] OR "fitness track*"[Title/Abstract] OR "smartphone*"[Title/Abstract] OR "smartwatch*"[Title/Abstract] OR "smart device"[Title/Abstract] OR "acceleromet*"[Title/Abstract] OR "motion sens*"[Title/Abstract] OR "actigraph*"[Title/Abstract])) |
| **Web of Science** | ((TS=(("machine learning" OR "deep learning" OR "neural net*" OR "reinforcement learning" OR "artifical intelligence")))  AND  TS=(("physical activ*" OR "physical fitness" OR walking OR "exercis*" OR "energy expenditure" OR "energetic arousal" OR "step*" OR "locomotive activ*" OR "ambulatory mov*" OR "activity intens*" OR "free-living condition" OR locomotion OR "activity pattern*" OR sport* OR "everyday activ*" OR "free-living activ*" OR "sedentar*" OR sitting OR "sedentary behav*" OR "body posture" OR "physical behav*" OR "physical inactiv*") ))  AND  TS=(("wearable" OR "activity monitor*" OR "activity track*" OR "fitness track*" OR "smartphone*" OR "smartwatch*" OR "smart device" OR "acceleromet*" OR "motion sens*" OR "actigraph*")) |
