# Supplementary material for: Applying AI in the Context of the Association Between Device-Based Assessment of Physical Activity and Mental Health: Systematic Review
Source: JMIR Mhealth Uhealth. 2025 Mar 6;13:e59660. doi: 10.2196/59660 (PMC11926455; doi:10.2196/59660)
Supplement: Multimedia Appendix 3 [file mhealth_v13i1e59660_app3.docx]

- Missing data management and data set balancing
  - 1 point if handling of missing data is described
  - 1 point if imbalanced data set problem is addressed
- Data Pre-processing
  - 1 point for mentioning steps
  - 1 additional point for explaining (necessity of) steps
- Definition of Outcomes
  - 1 point if intentional outcome is mentioned
  - 1 additional point if intentional outcome is formulated in hypotheses
- Sample Description and Eligibility Definition
  - 1 point for basic information in text (N, age, gender)
  - 1 additional point for supplementary info (ethnicity, group type, etc.)
- Representativeness
  - 0-2 points for non to fully fulfilled item (2 points only when developed model was tested with an independent dataset)
- Justification of sample size
  - 0-2 points for non to fully fulfilled item
- Feature Extraction/Selection
  - 1 point if features are described/listed and how they are extracted
  - 1 point if feature selection is described (feature evaluation process)
- Justification of Classifier Performance
  - 1 point for description of classifier performance
  - 1 point for explanation of classifier performance
- Best feature performance eval
  - 1 point for mentioning best features
  - 1 point for examining/explaining feature importance
