# Supplementary material for: Applying AI in the Context of the Association Between Device-Based Assessment of Physical Activity and Mental Health: Systematic Review
Source: JMIR Mhealth Uhealth. 2025 Mar 6;13:e59660. doi: 10.2196/59660 (PMC11926455; doi:10.2196/59660)
Supplement: Multimedia Appendix 4 [file mhealth_v13i1e59660_app4.docx]

**Supplementary file.** Overview of all included studies and respective extracted information.

| No | Author (year; location) | Sample Size (mean age±SD or range [yrs]; % males, population) | Study duration [days] (type; environment) | Mental health outcome (instrument) | Accelerometery hardware (placement) | Machine learning algorithms used^1^ | Study conclusion | QA |
| --- | --- | --- | --- | --- | --- | --- | --- | --- |
| 1 | Ahmed et al [30] (2022; United Arab Emirates) | 87 (NR^2^; NR; volunteers) | 5 (observational, free living) | Depression (BDI-II, momentary ratings) | Psychorus (wrist) | CB, LR, SVM, kNN, LGB, RF, XGB | Classifying between moderately and severely depressed individuals using wearable-derived physiological data | 7 |
| 2 | Asare et al [31] (2022; Finland) | 54 (NR; NR; none) | 30 (observational; free living) | Depression (DASS, CMA) | Oura ring, smartphone (finger, NR) | SVM, RF, XGB, kNN, LR | Understanding depression and the potential of mobile sensor data to augment depression diagnosis and monitoring | 7 |
| 3 | Booth et al [32] (2022; USA) | 597 (34.4±9.4; 58%; NR) | 56 (observational, natural work environment) | Stress (momentary ratings) | Garmin Vivosmart 3 (wrist) | EN, RF, MLP, GRU, LSTM | Automated perceived stress inference | 8 |
| 4 | Can [33] (2022; Turkey) | 14 (20-25; 64%; students (college)) | <1 (psychological experiment; laboratory) | Stress (PSS-14, TSST) | Empatica E4 (wrist) | LR, kNN, MLP, SVM, RF | Differentiation of relaxed, stressful and physically active states through machine learning | 6 |
| 5 | Can et al [34] (2019; Turkey) | 21 (20; 86%; students (high school and university)) | 9 (observational; laboratory) | Stress (momentary ratings) | Samsung Gear S, Empatica E4 (wrist, wrist) | SVM, RF, LR, kNN | Discrimination of content stress, high cognitive load and relaxed time by using different machine learning methods | 5 |
| 6 | Chakraborty et al [35] (2019; Republic of Korea) | 15 (27.5 ± 2.4; 80%; graduate students) | <1 (psychological experiment; laboratory) | State of mind (TSST) | RespiBAN Professional, Empatica E4 (chest, wrist) | Multi-channel CNN | Detection of the State of Mind using physiological signals from wearable devices | 8 |
| 7 | Choi et al [36] (2021; Republic of Korea) | 1552 (NR; NR; NR) | 7 (observational; NR) | Depression (PHQ-9) | ActiGraph GTX3 (NR) | XGB, SVM, MLP, LR | Classifying depression levels using machine learning | 9 |
| 8 | Doryab et al [37] (2019; USA) | 160 (NR; NR; college students) | 112 (observational; free living) | Loneliness (UCLA loneliness scale) | Fitbit Flex 2 (non-dominant wrist) | GB, LR | Using ubiquitous smartphone and wearable sensors to passively detect loneliness | 10 |
| 9 | Fukazawa et al [38] (2019; Japan) | 20 (20-24; 75%; healthy, no history of mental illness) | 30 (observational; free living) | Stress (STAI) | Smartphone (NR) | XGB, RF | Extract co-occurring combination of a user’s environment, real-world behavioral, and online behavioral features | 8 |
| 10 | Garcia-Ceja et al [39] (2018; Norway) | 55 (NR; NR; patients and control) | 12.6 (observational; free living) | Depression (MADRS) | Actigraph AW4 (right wrist) | RF, DNN | Classification of depressed and nondepressed patients using motor activity | 7 |
| 11 | Gwak et al [40] (2018; USA) | 53 (71.58 ± 11.36; 47%; patients and control) | 1 (observational; clinical setting) | Mild cognitive impairment (NINCDS-ADRDA, Petersen criteria) | Samsung Gear Live (non-dominant wrist) | LR, RF, Extra Trees | Classification between MCI and cognitively healthy adults and providing an algorithm to support diagnosis | 7 |
| 12 | Hart et al [41] (2021; Germany) | 158 (41.6 ± 10.9; 34%; employees from different professions) | 21 (observational; free living) | Psychological state (MDBF) | Smartphone (hand) | RF, penalized GLM | Infer a sizable proportion of variance in self-reported states via personalized machine learning models | 9 |
| 13 | Hashmi et al [42] (2020; Pakistan) | 40 (25.2 ± 5.9; 65%; NR) | NR (observational; laboratory setting) | Emotions (NR) | Smartphone [ MPU-6500] (chest) | RF, SVM | Estimate emotions from human gait data | 7 |
| 14 | Husom et al [43] (2022; Greece) | 35 (45 ± 13; 11%; NR) | 7 (observational; free living) | Fatigue (Fatigue Assessment Scale) | Fitbit Charge 5 (wrist) | DT, RF, XGB, kNN, FCNN, LSTM | Predicting the FAS-score | 5 |
| 15 | Jacobsen, Feng [44] (2022; USA) | 264 (20-39; 53%; none) | 7 (observational; free living) | Generalized Anxiety Disorder (Composite international diagnostic interview (CIDI, version 2.1)) | ActiGraph AM-7164 (hip) | XGB | Assessing GAD symptom severity using passive movement data | 4 |
| 16 | Jacobson et al [26] (2020; USA) | 59 (19.8 ± 2.4; 49%; college students) | 14 (observational; free living) | Social Anxiety Disorder (SIAS, DASS-21, PANAS) | Smartphone (NR) | XGB | Utilize passive smartphone sensor data to improve ability to detect behavioral indicators of problematic pathology | 6 |
| 17 | Jakobsen et al [45] (2020; Norway) | 23+32 (42.8 ± 11.0; 57%; bipolar and unipolar patients + healthy controls) | 14 (observational; clinical setting) | Depression (DSM-IV, MADRS) | Actigraph (wrist) | RF, (weighted) DNN, weighted CNN | Illustrate abilities of various machine learning algorithms to discriminate between depressed patients and healthy controls | 2 |
| 18 | Kilimci et al [46] (2019; Turkey) | 15 (NR; NR; NR) | 365 (observational; free living) | Mood (momentary ratings) | Apple iWatch, Vestel smart watch (wrist, wrist) | SVM, DT, RF, FFNN, RNN, CNN, LSTM, ANN | Develop a model for emotional analysis of users | 2 |
| 19 | Kim et al [47] (2019; Republic of Korea) | 47 (78 ± 5,24; 6%; had less than high school education (42/47, 89%), and had a moderate socioeconomic status (35/47, 74%)) | 14 (observational; free living) | Depression (Short Geriatric Depression Scale (SGDS-K) and the Hamilton Depression Rating Scale (K-HDRS)) | Actiwatch Spectrum PRO (non-dominant wrist) | LR, DT, BT, RF | Classifying depression groups based on EMA and actigraphy data | 8 |
| 20 | Lekkas et al [27] (2022; USA) | 75 (12-20; 0%; patients and controls (AN (N = 44) and healthy controls (N = 31))) | 1 (observational; free living) | Anorexia nervosa (EDI‐2, PANAS‐X) | SOMNOwatch (non-dominant wrist) | XGBT | Prediction of AN and HC emotional state change via longitudinally and passively measured PA | 7 |
| 21 | Lietz et al [48] (2019; USA) | 2 (NR; NR; NR) | NR (observational; NR) | Mood (momentary ratings) | Fitbit Ionic (wrist) | DT | Predicting mood given heart rate and activity data | 1 |
| 22 | Luo et al [49] (2020; Switzerland) | 28 (26-55; 51%; recovering from jet lag) | 7 (observational; free living) | Fatigue (PhF, MF, VAS, PelP) | Everion (non-dominant wrist) | RF, CNN | Using multimodal digital data to inform, quantify, and augment subjectively captured fatigue | 9 |
| 23 | Magal et al [50] (2022; Israel) | 129 (18-45; 0%; none) | 1+7 (Experiment and observation; free living) | Stress (Trier Inventory for Chronic Stress) | Fitbit Charge 3 (wrist) | SVM | Integration of daily life indicators could improve our understanding of chronic stress and its impact | 11 |
| 24 | Masud et al [51] (2020; Bangladesh) | 33 (24 ± 5; 58%; students) | 77 (observational; free living) | Depression (QIDS-SR16) | Smartphone (NR) | SVM, kNN, ANN | Cost-effective solution for identifying depression without invading personal space | 9 |
| 25 | Masud et al [28] (2022; Bangladesh) | 33 (24 ± 5; 58%; none) | 77 (observational; free living) | Depression (PHQ-9) | Smartphone (NR) | LSTM-RNN | Cost-effective solution for identifying depression without invading personal space | 9 |
| 26 | Maxhuni et al [52] (2021; Denmark) | 30 (37.46 ± NR; 60%; none) | 56 (observational; free living) | Stress (momentary ratings (Oldenburg Burnout Inventory and POMS)) | Samsung Galaxy S3 mini (NR) | DT | Extensive analysis related to stress using information derived from smartphones | 7 |
| 27 | Narziev et al [53] (2020; Republic of Korea) | 20 (NR; NR; NR) | 28 (observational; free living) | Depression (PHQ-9, DSM-5 (SCID-5), BDI-II, STAI) | Smartphone, Samsung Gear S3 Frontier  (NR, wrist) | SVM, RF | Short term depression detection using every day mobile devices | 7 |
| 28 | Olsen, Torresen [54] (2016; Norway) | 10 (NR; NR; NR) | NR (observational; NR) | Emotions (mood) | Smartphone (pocket) | DT, SVM, MLP | Classifying emotions using accelerometer data from a smartphone | 5 |
| 29 | Padmaja et al [55] (2019; India) | 35 (NR; NR; none) | 60 (observational; free living) | Stress (PSS) | Fitbit (wrist) | DT | Extensive analysis related to stress using information derived from smartphones | 0 |
| 30 | Padmaja et al [56] (2018; India) | 10 (28 – 45; NR; adults working in IT and other sectors) | 300 (observational; free living) | Stress (PSS) | Fitbit (wrist) | LR | Physical activity acts as a de-stress agent on human stress | 4 |
| 31 | Quiroz et al [57] (2017; UK) | 50 (23.18 ± 4.87; 14%; students (university)) | <1 (observational; laboratory) | Emotions (PANAS) | Samsung Gear 2 (left wrist) | RF, LR | Determining the emotions of individuals based on the accelerometer data from a smart watch | 6 |
| 32 | Ross et al [58] (2023; USA) | 100 (NR; NR; NR) | NR (observational; free living) | Depression (PHQ-8) | Smartphone (while typing) | RF, GB, DNN | Processing accelerometer data to augment prediction of changes in depression severity | 7 |
| 33 | Rozet et al [29] (2019; USA) | 77 (31.62 ± 9.42; 44%; none) | 356 (observational; free living) | Stress (PSS) | Fitbit (wrist) | IDT, RF, GB, RNN, EN, NN | Predicting a future stress rating | 7 |
| 34 | Rykov et al [59] (2021; Singapore) | 267 (32.8 ± 8.6; 37%; working adults) | 14 (observational; free living) | Depression (PHQ-9) | Fitbit Charge 2 (wrist) | XGB | Some biomarkers based on data from consumer wearables could indicate increased risk of depression | 15 |
| 35 | Sadeghi et al [25] (2022; USA) | 99 (45.5 ± 10; 83%; veterans) | 7 (observational; clinical setting (seven Project Hero’s United Healthcare Ride 2 Recovery challange)) | PTSD (Hyperarousal event timestamps) | MOTO 360 Gen 1 or Gen 2, Apple Watch series 3 or 4 (wrist, wrist) | RF, XGB, LR, non-linear SVM | Using Machine learning algorithms to predict PTSD using smartwatch rate and accelerometer data | 10 |
| 36 | Saito et al [60] (2022; Japan) | 4612 (45.9 ± 9.1; 71%; none) | 90 (observational; free living) | All (NR) | Fitbit (wrist) | XGB | Using Machine learning to build a predictive model that used sleep and activity data to establish the criteria leading to mental illness onset | 11 |
| 37 | Sanchez et al [61] (2023; Mexico) | 57 (NR; 42%; desk jobs) | 5 (observational; free living) | Stress (momentary ratings) | Fitbit (wrist) | kNN, NB, RF, J48, AB | Recognizing behavioral patterns and infers stress | 6 |
| 38 | Sa-nguannarm et al [62] (2023; Republic of Korea) | 15 (NR; NR; NR) | <1 (observational; laboratory) | Stress (Trier Social Stress Test) | RespiBAN, Empatica E4 (chest, chest) | LSTM | Proposed HAR model recognizes stress levels | 4 |
| 39 | Sevil et al [63] (2020; USA) | 34 (NR; NR; none) | <1 (observational; laboratory) | Stress (STAI) | Empatica E4 (wrist) | kNN, SVM, DT, NB, DL | Using a wristband device for monitoring the factors contributing to an elevated risk of acute events | 4 |
| 40 | Shah et al [64] (2021; USA) | 14 (21.6 ± 2.8; 30%; students (college), depression symptoms) | 30 (observational; free living) | Depression, mood (PHQ-9, momentary ratings) | Samsung Galaxy wristwatch (wrist) | RF, GB, AB, EN, SVM | Generate a personalized machine learning pipeline to predict depression mood | 5 |
| 41 | Spulber et al [65] (2022; Sweden) | 17 (NR; NR; patients (ongoing major depressive episode, with MADRS ≥ 20, resistant to selective serotonin reuptake inhibitor (SSRI) treatment in an adequate dose for at least 4 weeks)) | 2 (observational; clinical setting) | Depression (DSM-IV, MADRS) | GENEActiv Original actigraph, Actiwatch 2 (wrist, wrist) | NR | Symptom severity can be predicted by analyzing the subject`s activity | 3 |
| 42 | Sultana et al [66] (2020; Canada) | 18 (NR; NR; students) | 3-9 (observational + exploratory; free living) | Emotions (momentary ratings) | Smartphone, Smartwatch (NR, wrist) | LR, RF, XGB, CB, MLP | Detecting emotional transitions and states from daily contextual information using machine learning | 8 |
| 43 | Sükei et al [67] (2021; Spain) | 943 (41 (18-77); 37%; outpatients (recruited from community clinics)) | 30 (Experiment + observational; free living) | Emotions (momentary ratings) | NR (NR) | SVM, LR, RF, MLP, RNN, LSTM, GRU | Designing machine learning models for predicting emotional states from mobile sensing data | 7 |
| 44 | Tazawa et al [68] (2020; Japan) | 45+41 (52.1 ± 13.2, 69.1 ± 14.2; 53%; depression inpatients and outpatients + healthy controls) | 60 (observational; free living, clinical setting) | Depression (HAMD, MADRS, YMRSm BDI-2, PSQI) | Silmee W20 (NR) | XGB, SVM, RF | Utilized machine learning to predict the presence and severity of depression state | 12 |
| 45 | Umematsu et al [69] (2020; Japan/USA) | 39+201 (20-50; NR; workers, students) | 30 (observational; free living) | Stress, mood (momentary ratings (stress, mood, and health scores), PSS) | Empatica E4, Affectiva  Q (wrist, wrist) | RF | Forecast for workers daily well-being scores | 4 |
| 46 | Umematsu et al [70] (2019; USA) | 142 (NR; NR; students (college)) | 8 (observational; free living) | Stress, happiness (momentary ratings (Stress, health and happiness scores) | Affectiva Q (wrist) | LSTM, SVM, LR | Forecast of tomorrow`s well-being using daytime-only physiology data from wearable sensors | 2 |
| 47 | Umematsu et al [71] (2021; USA) | 142 (NR; NR; students (college)) | 8 (observational; free living) | Stress (momentary ratings) | Affectiva Q (wrist) | LSTM, SVM, LR | Forecasting of stress using data collected in an unobtrusive way | 1 |
| 48 | Yan et al [72] (2020; USA) | 212+48 (NR; NR; hospital workers + students) | 70 (observational; free living) | Affect (PANAS-Short, PAM) | Fitbit Charge 2, smartphone (wrist, NR) | RF, XGB, MERF | Estimating daily self-reported affects from sensor-generated data | 5 |
| 49 | Zebin et al [73] (2019; UK) | 200+63 (NR; NR; patients + controls) | 7 (observational; free living) | SMI (momentary ratings) | Axivity AX3 (NR) | DNN, RF | Classifying a serious mental illness participant group from a control group using activity tracking data | 7 |
| ^1^ AB=Ada Boost, ANN=Artificial Neural Network, BT=Boosted Tree, CB=Cat Boost, CNN=Convolutional Neural Network, DNN=Deep Neural Network, DT=Decision Tree, EN=Elastic Net, FCNN=Fully-Connected Neural Network, FFNN=Feed-Forward Neural Network, GB=Gradient Boosting, GLM=Gated Recurrent Network, kNN=k-nearest neighbour, LGB=Light Gradient Boosting, LR=Linear Regression, LSTM=Long Short-Term Memory, MERF=Mixed Effect Random Forest, MLP=Multi-Layer Perceptron, NB=Naïve Bayes, RF=Random Forest, RNN=Recurrent Neural Network, SVM=Support Vector Machine, XGBT=Extreme Gradient Boosted Tree  ^2^ NR=Not reported | | | | | | | | |
