# Supplementary material for: Applying AI in the Context of the Association Between Device-Based Assessment of Physical Activity and Mental Health: Systematic Review
Source: JMIR Mhealth Uhealth. 2025 Mar 6;13:e59660. doi: 10.2196/59660 (PMC11926455; doi:10.2196/59660)
Supplement: Multimedia Appendix 5 [file mhealth_v13i1e59660_app5.docx]

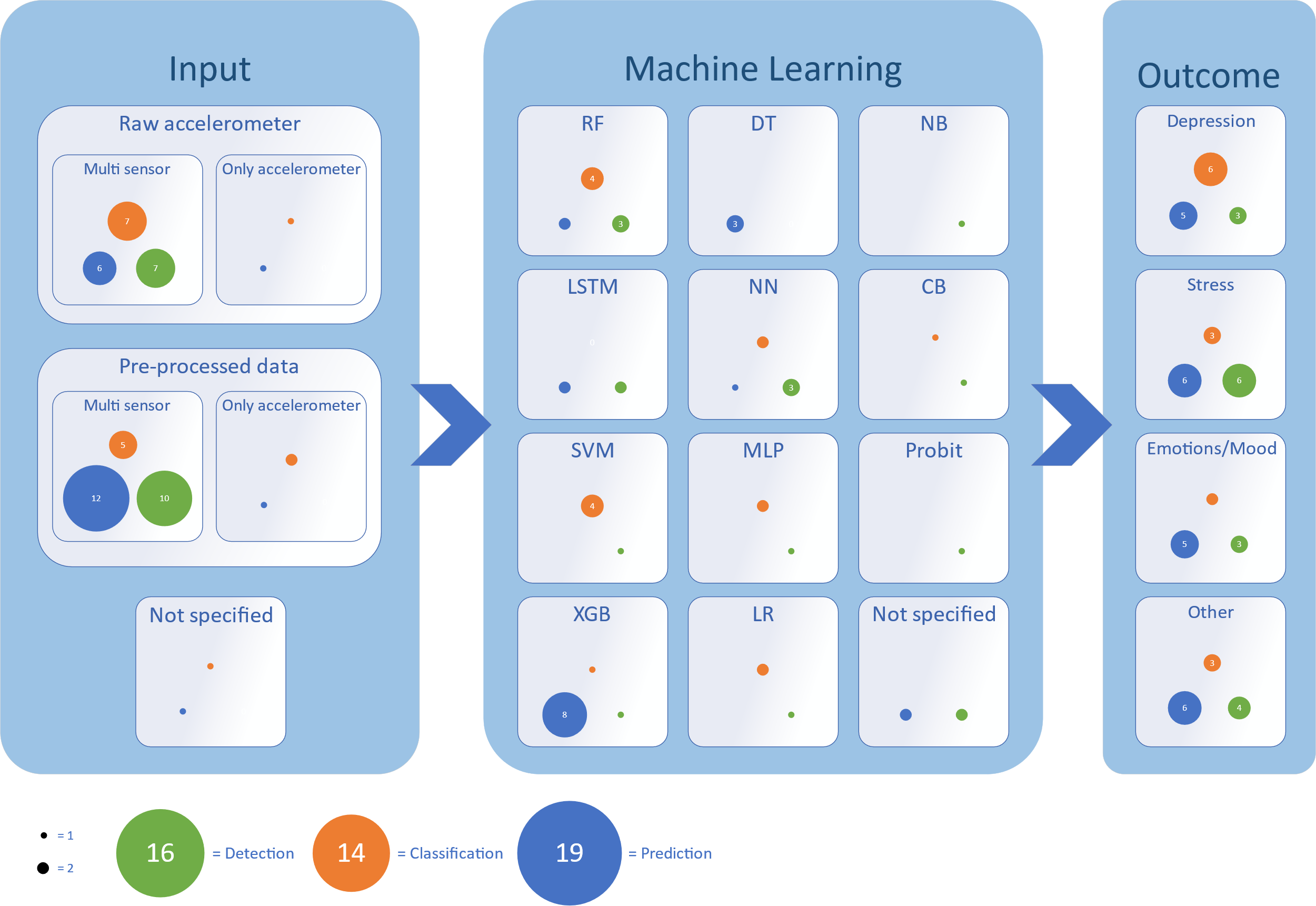


**Figure S1.** Overview of the input, machine learning, and outcome differentiated by the study aims. Notably, some studies appear multiple times due to different input configurations. RF=Random Forest, DT=Decision Tree, NB=Naïve Bayes, LSTM=Long Short-Term Memory, NN=Neural Network, CB=Cat Boost, SVM=Support Vector Machine, MLP=Multi-Layer Perceptron, XGB=Extreme Gradient Boost, LR=Linear Regression.


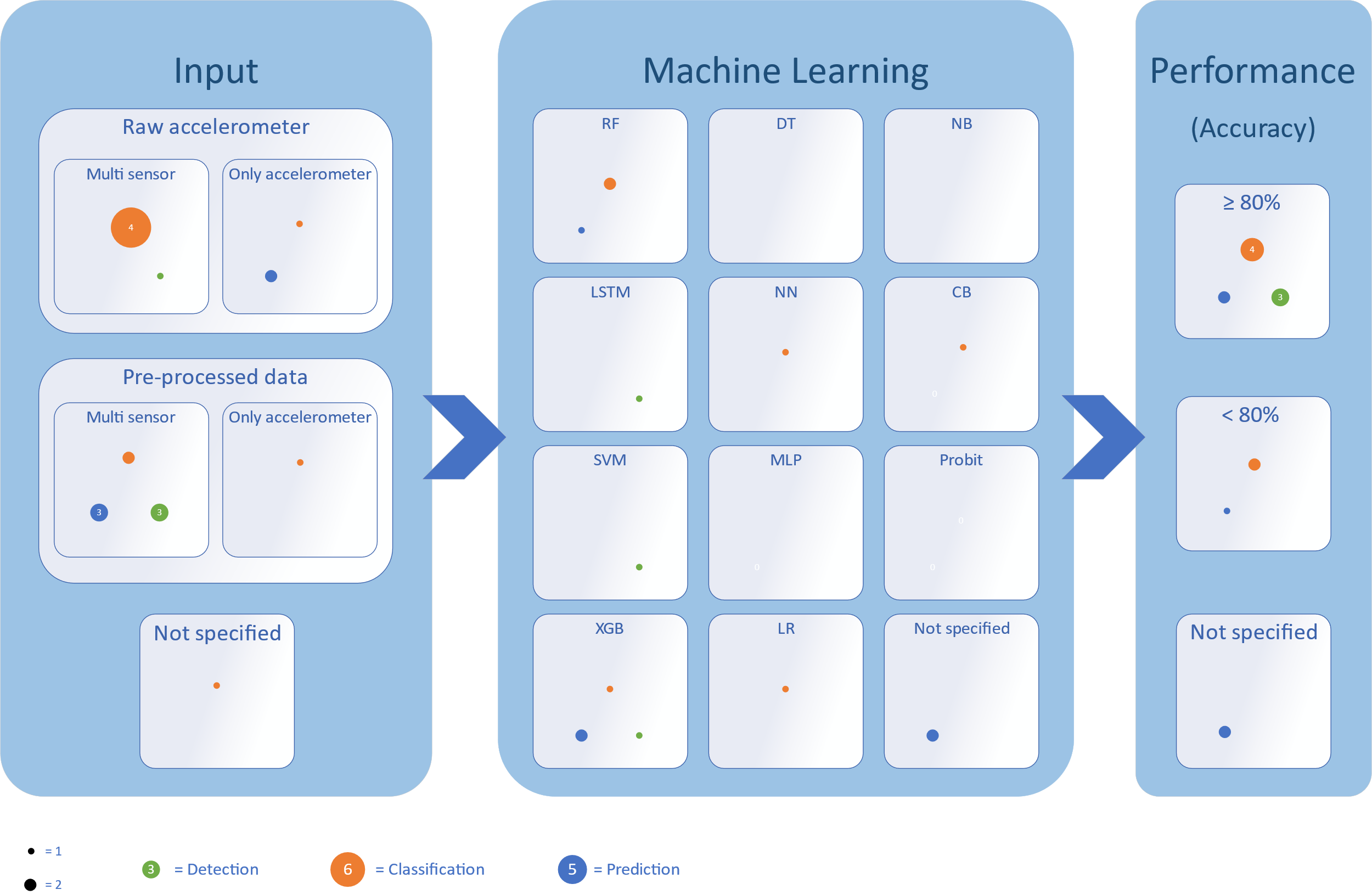


**Figure S2**. Overview of the input, machine learning, and performance approaches differentiated by the study aims for articles studying depression. Notably, some studies appear multiple times due to different input configurations. RF=Random Forest, DT=Decision Tree, NB=Naïve Bayes, LSTM=Long Short-Term Memory, NN=Neural Network, CB=Cat Boost, SVM=Support Vector Machine, MLP=Multi-Layer Perceptron, XGB=Extreme Gradient Boost, LR=Linear Regression.


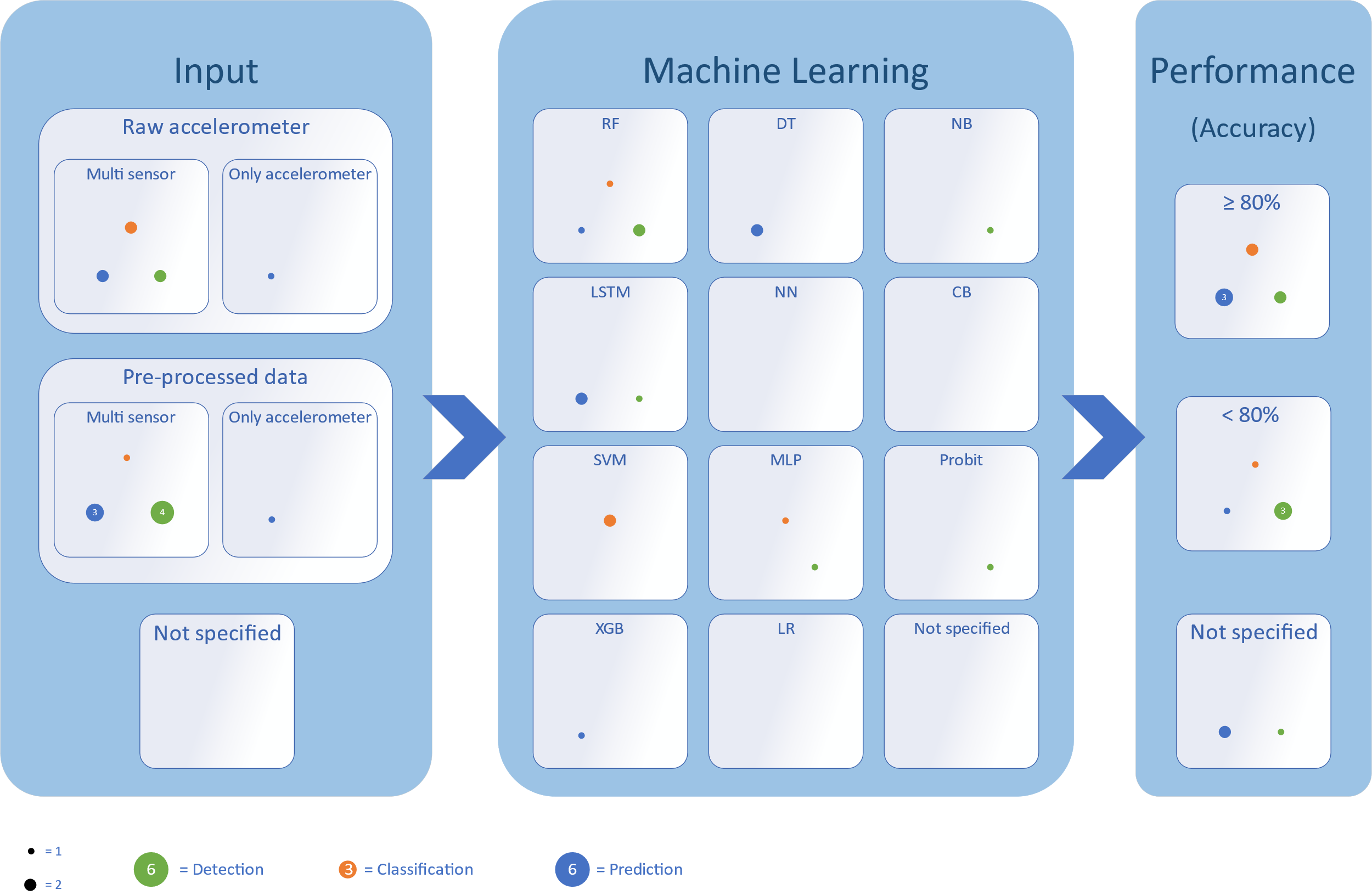


**Figure S3.** Overview of the input, machine learning, and performance approaches differentiated by the study aims for articles studying stress. Notably, some studies appear multiple times due to different input configurations. RF=Random Forest, DT=Decision Tree, NB=Naïve Bayes, LSTM=Long Short-Term Memory, NN=Neural Network, CB=Cat Boost, SVM=Support Vector Machine, MLP=Multi-Layer Perceptron, XGB=Extreme Gradient Boost, LR=Linear Regression.
